# Gender Differences in the Psychopathology of Mixed Depression in Adolescents with a Major Depressive Episode

**DOI:** 10.2174/1570159X20666221012113458

**Published:** 2023-05-12

**Authors:** Massimo Apicella, Giulia Serra, Maria Elena Iannoni, Monia Trasolini, Gino Maglio, Elisa Andracchio, Stefano Vicari

**Affiliations:** 1 Department of Neuroscience, Child Neuropsychiatry Unit, Bambino Gesù Children’s Hospital, IRCCS, Rome, Italy;; 2 Department of Life Sciences and Public Health, Catholic University, Rome, Italy

**Keywords:** Adolescent, child, juvenile, bipolar, major depressive, mixed depression, gender, sex

## Abstract

**Background:**

Gender differences have been reported in the severity and psychopathological features of major depressive disorders among adults but are poorly reported in adolescent samples.

**Objective:**

This study aimed to examine gender differences in the psychopathology of mixed depression among adolescents.

**Methods:**

We analyzed 341 outpatients with the current major depressive episode (MDE) retrospectively to identify patients with DSM-5 MDE with mixed features. We compared examiner-rated depressive and (hypo)manic symptoms and self- and parent-reported symptoms between sexes.

**Results:**

We identified 76 patients with an MDE with mixed features (67.1% females, 32.9% with bipolar disorder). Depression severity was significantly greater in females *versus* males (CDRS-R total score 56.2 *vs*. 48.2, p = 0.014). Depressive symptoms were significantly and independently found to be more severe among females in a logistic regression model, including excessive fatigue (OR 1.68; p = 0.025), low self-esteem (OR 1.67; p = 0.04), excessive weeping (OR 1.62; p = 0.021), and CBCL AAA index (OR 1.04; p = 0.015). None of the depressive symptoms scored greater in males. Males had higher levels of motor activity (2.12 *vs*. 1.69; p = 0.048) and pressured speech (1.80 *vs*. 1.24; p = 0.004). Self-rated anxiety (69.3 *vs.* 56.8, p = 0.047) and CBCL AAA index (207 *vs.* 189; p = 0.007) were higher in females.

**Conclusion:**

Adolescent depression with mixed features is more severe in women, with a higher expression of core affective symptoms and excessive fatigue. While in males, slightly higher levels of psychomotor activation are reported, in females, emotional dysregulation and excessive weeping may subtend a difference in a broader spectrum of mixed features.

## 1 INTRODUCTION

Juvenile major affective disorders are prevalent psychiatric illnesses with a high risk of morbidity and disability that severely limit psychosocial functioning and reduce the quality of life, with an increased risk of fatal outcomes for suicide and other comorbid medical conditions [1]. Bipolar disorder and major depression are also leading contributors to general health burdens and decrease longevity associated particularly with suicide and ischemic heart disease [2].

In 2008, the World Health Organization ranked major depression third as a cause of burden of disease worldwide and projected that it will rank first by 2030 [3]. The Global Burden of Disease Study in 2010 identified depression as the second leading cause of years lived with disability (YLDs), accounting for about 10% of global YLDs and ranking among the leading causes of school absence [2].

The lifetime prevalence of depression in a community sample of more than 10,000 adolescents aged 12 to 18 years was 11% for major depression, more than a quarter of which (3%) was accounted for by severe depression [4]. Prevalence of depression increases significantly from childhood to late adolescence, especially among females; it peaked at 13% for 15-17-year-old females, including 5% with severe depression. Severe depression is associated with a greater risk of psychiatric comorbidity, suicide and functional impairment than mild or moderate forms [4].

Several epidemiologic studies demonstrate a higher prevalence of depression in women than men [5]. Different neurobiological, psychosocial, and methodological factors have been invoked to explain this difference [6]. The difference in the incidence of depression is evident after puberty and peaks in late adolescence [7], with a significantly greater incidence among females than males, both for sub-syndromal and fully expressed major depression [8].

Studies on the prevalence of bipolar disorder report a less marked difference between genders, finding no relevant difference [9] or sometimes a greater prevalence in males [10].

Some differences have been reported in the phenomenology and course of bipolar disorder in adult women *versus* men, including a greater prevalence of a first depressive episode [11], more depressive episodes during their illness course [12], more mixed features in (hypo)manic episodes [13], more depressive episodes with psychotic features and a greater probability of receiving a diagnosis of personality disorder among women compared to men [14].

Several studies have observed differences in depressive symptoms among genders in adult populations, but a meta-analysis of adult studies concluded that those differences are of small entity [15]. More limited data are available on symptoms’ differences in bipolar depression in adults, with small evidence for a greater representation in women in terms of appetite and sleep alterations, concentration disturbances, worsening of symptoms in the morning, consummatory anhedonia and suicidal ideation [16]. Also, depression with atypical features is more common in women [17], while depression with melancholic features has a similar incidence among genders [10].

Evidence of gender difference in the psychopathology of depression in adolescence is more limited. Community studies reported a general greater severity of self-reported depressive symptoms among females [18], and a clinical study found more severe thoughts of guilt and failure, self-image dissatisfaction, depressive feelings, difficulties in concentrating, excessive fatigue and worse school functioning among adolescent females compared to males [19]. Some inconsistencies emerged between clinician and self-reported sleep and concentration difficulties. Some previous studies, on the other hand, did not find any significant difference in symptoms’ profiles [20-23].

Mixed depression in DSM-5 is defined as depression with associated classic manic symptoms. The DSM-5 criteria for the diagnosis of depression with mixed specifiers exclude “overlapping” symptoms, such as irritability and distractibility, which may be present primarily in depression. This construct has been criticized for leading to a restrictive definition of mixed states [24], whereas in adolescents, depression with irritability and possible counter-polar features is very common. According to DSM-5, mixed features are identified in about 10% of adult outpatients diagnosed with major depression, are more frequently identified in women, and are associated with a worse longitudinal course [25]. Considering a broader definition of depression with subthreshold hypomanic symptoms, mixed features are more common overall and retain a similar gender difference in the longitudinal course [25].

In pediatric populations, mixed features characterized by psychomotor agitation, crowded thoughts, and chronic irritability are frequently comorbid with anxiety, attention-deficit/hyperactivity, and conduct disorders and may represent a developmental subtype of early onset bipolar disorder [26]. Mixed features have been shown to delineate a distinct and more severe phenotype of depression in young patients [27]. Moreover, differences in comorbidities have been observed between adult women and men, with more anxiety and comorbid somatic symptoms among females and more impulsive/disruptive/conduct and substance use comorbid features among males [28].

Finally, a well-known gender paradox has been largely described in epidemiological studies on suicidal behavior, consistently reporting a greater prevalence of suicide attempts among women, but a significantly greater risk of suicide among men in all age groups [29-30], with an apparent greater increasing of this difference with age [31].

Sex differences in mixed features have been studied in hypomania in adults, finding significant gender differences in incidence and symptoms profile [32]. Hypomanic women have been reported to be more prone to experience mixed and depressive symptoms. While in men, the depression symptom scale tends to have higher scores during hypomanic states due to a more frequent increase in irritability and agitation; women are reported to experience mixed depressive symptoms, whose level increases with increasing hypomania severity, with the exception of most severe (hypo)manic states [32].

To our knowledge, no study has assessed gender differences in the psychopathology of mixed depression in adolescence. The aim of this study was to assess differences in depressive symptomatology between male and female juvenile patients diagnosed with a major depressive episode with mixed features and followed at the Mood Disorder Program of the Child and Adolescent Psychiatry at Bambino Gesù Children’s Hospital (OPBG) in Rome.

## 2 MATERIALS AND METHODS

### 2.1 Study Subjects

We analysed differences in depressive symptomatology between male and female juvenile patients diagnosed with a major depressive episode at the Mood Disorder Program of Bambino Gesù Children’s Hospital in Rome. The present sample was recruited from a Day Hospital for the assessment and treatment of early-onset mood disorders. Historical and prospective data obtained during clinical assessment and treatment were extracted from clinical records. Subjects are first routinely screened in general outpatient clinics and referred to the Day Hospital if presenting an illness severe enough to require assessment and treatment in a specialized psychiatric center. Possible referring sources include primary care doctors (pediatricians and general practitioners), secondary care (child psychiatrists in national healthcare centers) or the OPBG emergency department. Data were collected from January, 2012, to October, 2021.

Included subjects were children and adolescents aged from 6 to 18 years at evaluation, diagnosed with a major affective disorder, currently experiencing a major depressive episode of any level of severity. Categorical diagnoses were made clinically and confirmed with the Kiddie-Schedule for Affective Disorders and Schizophrenia for School-aged Children, Present and Lifetime version (K-SADS-PL) [33] following the DSM-5 criteria.

We included subjects diagnosed with a Major Depressive Episode (MDE) in the context of either a major depressive or a bipolar disorder. Subjects diagnosed with a persistent depressive disorder with intermittent major depressive episodes, showing a current major depressive episode, were also included.

We excluded patients with intellectual disability of moderate to profound severity, autism spectrum disorder and/or diagnosis of substance-induced mood disorders and/or mood disorders due to another medical condition according to DSM-5.

Parents/legal representatives of each patient provided written, informed consent at the clinic for potential research analysis and anonymous reporting of findings in aggregate form, in accordance with Italian legal and ethical requirements for clinical data. The study was conducted in accordance with the Declaration of Helsinki (1964).

To maximize the reliability of study data, records for each subject were reviewed, and required data were summarized in structured research forms, independently by two investigators (GS and MA), working to consensus with a third (GM) to resolve even minor differences.

### 2.2 Evaluation Procedure

Subjects were evaluated at the Day Hospital during at least 3 appointments providing a total of 9-10 hours of clinical assessment. All adolescents were assessed by an experienced child and adolescent psychiatrist and an experienced psychologist using the Schedule for Affective Disorders and Schizophrenia for School-Age Children-Present and Lifetime Version (K SADS-PL) [33]. A semi-structured, clinician-administered diagnostic interview was performed with both subjects and their parents or adult legal representatives and was used to confirm the primary diagnosis of mood disorder and assess comorbidities.

In addition, depressive and manic or mixed symptoms were rated by the same experienced clinicians using the Children Depression Rating Scale-Revised (CDRS-R) [34] and the K-SADS Mania Rating Scale (KMRS) [35], respectively. Also, during the assessment visits, the following standard rating scales were scored: Child Depression Inventory (CDI) [36] for self-rating of depressive symptoms, investigator-rated Clinical Global Assessment Scale (CGAS) [37] to evaluate the global function, Multidimensional Anxiety Scale for Children (MASC) [38] to assess self-rated anxiety features, and Child Behavior Checklist for Ages 6-18 (CBCL) [39]. Non-suicidal self-injurious acts, suicidal ideation and suicidal behaviors were evaluated with the Columbia Suicide Severity Rating Scale (C-SSRS) [40]. Details of the evaluation procedure and the psychopathological assessment used in this study have already been described in a previous report of our group [41].

Depression severity at evaluation, whether mild, moderate, severe with/without psychotic symptoms or in partial/complete remission, was defined both on clinical judgment according to DSM-5 and with CDRS-R cutoffs of a total score of 30 to 44 for mild, 45 to 54 for moderate, 55 or more for severe depression and 29 or less for partial/complete remission. Mixed features were defined upon clinical judgment according to DSM-5. In line with previous literature on depression with mixed features in adolescents, patients with a score of 3 or more at the first KMRS item (elation) were excluded [27]. All included patients had 3 or more KMRS items scores of 3 or more, indicating the presence of manic symptoms of clinical significance and variable severity.

### 2.3 Structured Psychopathological Assessment

The K-SADS-PL [33] is a semi-structured schedule to assess current and past psychopathological features and psychiatric disorders in juveniles aged 6 to 18 years according to criteria of the Diagnostic and Statistical Manual of Mental Disorders, Fifth Edition (DSM-5) [42]. Study participants and at least one parent or legal guardian were interviewed to support the collection of all available data *via* the K-SADS-PL, including the primary mood disorder and comorbidities. It has been used in previous studies in Italy, showing good interrater reliability for diagnosis in a population of children and adolescents with mood disorders with a mean k of 0.85 [43].

The Children's Depression Rating Scale-Revisited (CDRS-R) [34] is a semi-structured interview used to rate depressive symptoms for ages 6-18 years on 17 items (rated 0 to 3, 4, 5, 6 or 7) with a raw score of 18-120 (given by the sum of the scores at single items and considered positive at scores >30). The assessment explores 17 scales: school dysfunctions, difficulties in having fun, difficulties in interpersonal relationships, sleep disorders, appetite disorders, excessive fatigue, psychosomatic complaints, irritability, excessive guilt, low self-esteem, depressive feelings, morbid ideas, suicidal ideation, excessive crying, reduced facial expressions, slow speech, and motor hypoactivity.

The Kiddie-SADS Mania Rating Scale (KMRS) [35] is a structured interview used to rate manic symptoms for ages 6- 18 years on 14 items, with a total score of 1-68 (given by the sum of the scores [0-6] for single items minus 13 and considered positive at scores ≥ 12). Symptoms rated in the KMRS are the following: euphoria and expansiveness, irritability and anger, mood lability, reduced need for sleep, crowded thoughts, increased energy, increased activities, motor hyperactivity, grandiosity, rapid or pressured speech, distractibility, impaired judgment, hallucinations, and delusions. A total score of 12 or more is considered indicative of significant manic symptoms; single items’ scores of <2 are considered normal, 2-3 borderline, and >3 as clinically significant [35].

C-GAS is used to assess global functioning [37]. The Columbia Suicide Severity Rating Scale (C-SSRS) is used to evaluate suicidal ideation and has been validated for ages of ≥12 years. The scale assesses individual levels of suicidal ideation and behaviours. Screening version-recent is administered to all patients; if a score ≥3 is recorded, the lifetime/recent version is administered [40].

Suicidal behavior is defined according to Posner (2014) [44]. A suicide attempt is defined as any self-harming behavior resulting in any damage with non-zero intent to die, declared by the patient or evident from documented circumstances. An interrupted attempt is defined as a behavior inevitably leading to a suicide attempt, interrupted by a person or external circumstance before resulting in any damage. Self-interrupted/aborted attempt is defined as a behavior inevitably leading to a suicide attempt, interrupted by the individual autonomously before resulting in any damage. Preparatory behavior is defined as any act which is prepared for the imminent performance of a suicide attempt, including access to a specific method and preparation for the perspective of own personal death. For the purposes of the present study, we defined the presence of “suicidal behavior” in the last 3 months before evaluation or lifetime as the presence of at least one suicidal attempt.

Non-suicidal self-injury (NSSI) in the last 3 months before evaluation or lifetime was systematically indagated and accurately differentiated from suicidal behavior, as defined in section III of DSM-5 [42].

The Italian version of the Child Behavior Checklist for ages 6-18 years (CBCL-6-18) [39] was completed by the proband and caregivers to rate behavioral and emotional problems in the study subjects. This extensively used tool provides scores with three behavior rating scales that address internalizing symptoms, externalizing symptoms, and total behavioral problems. Sub-items of these three scales include eight syndromal scales (withdrawn-depressed, somatic complaints, anxious-depression, social problems, thought problems, attention problems, rule-breaking behavior, and aggressive behavior). An overall “AAA” CBCL profile was calculated by summing scores for attention problems, aggression and anxious-depressed syndromal scales. This score is reported to be indicative of Deficient Emotional Self-Regulation (DESR), at scores of 180-210 (SD of 1-2), and as meeting criteria for a Dysregulation Profile (DP) at a score of >210 (>2 SD) for the sum of the 3 syndromal scale scores [45].

Children Depression Inventory 2 (CDI 2) [36], one of the most used measures of self-reported depressive symptoms in infancy, was administered to patients aged 7 to 17 and their parents. The self-report form is a 28 items assessment, while the parent report has 17 items exploring core features of depression.

Multidimensional Anxiety Scale for Children 2 (MASC 2) [38] was administered to patients aged 8 to 17 and their parents to assess for self-reported anxiety symptoms. It consists of 50-item modules that provide T-scores for six scales and four subscales considering emotional, physical, cognitive, and behavioural symptoms of anxiety. CDI 2 and MASC 2 were administered in 2018 after availability in the Italian version.

We further administered Affective Reactivity Index (ARI) [46], both in self-report and parent-report form, in 2017 to explore reported features of irritability.

### 2.4 Statistical Analysis

Categorical variables are presented as numbers and percentages. Continuous variables are presented as mean and standard deviation (SD). The association of categorical variables has been tested with Pearson’s or Fisher’s test, as appropriate. Differences between means of continuous variables have been tested with the Student’s t-test for unpaired variables. Correlation between continuous variables has been tested with Spearman’s Rho.

We further evaluated factors identified preliminarily as possibly associated with the female sex (at p<0.05, two-tailed), using multivariate logistic regression modeling to compute odds ratios (OR) with their 95% confidence intervals.

A p-value of 0.05 or less has been considered indicative of significance. Analyses were conducted with Microsoft Office 365 - Excel and IBM SPSS Statistics V26 software.

## 3 RESULTS

### 3.1 Subjects Characteristics

In the study period, 481 consecutive patients were evaluated, and 341 were diagnosed with a current major depressive episode. Clinician-administered rating scales were available for 309 patients, and 76 (24.6%) of them were diagnosed with a major depressive episode with mixed features.

Of the 76 subjects diagnosed with mixed depression, 25 were males, and 51 were females. Sixty-two patients (94.7%) were Caucasian, 2 were Middle-eastern, and 2 were Hispanic. There was no significant difference in the rate of patients of Caucasian ethnicity between sexes (96% of males and 94.1% of females, chi-square = 0.119, p = 0.73). The mean age at evaluation was significantly higher in females (14.9 *vs*. 13.5; p = 0.022, Table **1**). The mean level of education grade was 8.18 (SD 2.50, range 1^st^ to 12^th^ grade), with a mean of 7.2 (SD 2.94) in males and 8.67 (SD 2.12) in females (t = -2,48 p = 0.032).

Overall, more than half of the participants had a CDRS-R total score higher than 55, indicating high severity of depressive symptoms. There were significantly more female participants in the “moderate” (33.3% *vs* 24.0%, p = 0.003) and “severe” (58.8% *vs.* 36.0%, p = 0.003) severity group, defined by CDRS-R total score cutoffs of ≥ 55 and 45- to 54, respectively (Table **1**).

Diagnosis of bipolar disorder was made in 32.9% of the sample, with a non-significant trend of more diagnoses of bipolar disorder in males (40.0% *vs*. 29.4%; p = 0.422, Table **1**).

The most frequent comorbidities were anxiety disorders (25%), followed by learning disabilities (10.5%), eating and substance use disorders (6.57% each), ADHD (5.3%), oppositional defiant disorder, obsessive-compulsive disorder, and tic disorders (2.6% each). No patients had comorbid autism spectrum disorder. There were no significant differences in comorbidities between males and females (Table **1**).

More than 50% of the participants reported active suicidal ideation (52.0% of males *vs*. 64.7% of females, p = 0.28); the C-SSRS screening version mean score was 1.36 points in males and 2.25 in females (p = 0.48). At least one suicidal attempt in a lifetime was reported by 8.00% of males and 25.5% of females (p = 0.072). Non-suicidal self-injury (NSSI) was also common, reported in 36% of males and 52.9% of females (p = 0.16). One or more episodes of admission in a psychiatry ward were reported in 19.4% of females and 4% of males (p = 0.089).

### 3.2 Manic and Depressive Symptoms in Male *versus* Female Adolescents - Univariate Analysis

Depression severity measured with the CDRS-R total score was significantly greater in females than males (56.2 *vs.* 48.2, p = 0.014, Tables **1** and **2**).

Scores for some depressive symptoms were significantly greater in females than males, including excessive weeping (3.78 *vs.* 2.20; p = 0.001), excessive fatigue (3.94 *vs*. 2.76; p = 0.003), low self-esteem (4.51 *vs*. 3.60; p = 0.014), depressive feelings (4.75 *vs*. 3.80; p = 0.015), and suicidal ideation (3.16 *vs.* 2.20; p = 0.029) (Table **2**). None of the depressive symptoms were reported to be greater in males (Table **2**).

Manic symptoms measured with the K-MRS total score had a mean score of 13.12 in males and 11.29 in females (p = 0.052). The K-MRS cutoff of “≥ 12” for clinical significance of manic symptoms individuated 60% of males and 39.2% of females in our cohort (p = 0.088).

Some manic symptom scores were significantly greater in males versus females, including increased motor activity (2.12 *vs.* 1.69; p = 0.048) and pressured speech (1.80 *vs.* 1.24; p = 0.004).

### 3.3 Univariate Analysis of Self-rated Symptoms in Males *versus* Females

Anxiety symptoms, as measured by MASC total score rated by parents, were slightly greater among females than males (69.3 *vs.* 56.8, p = 0.047, Table **2**), as well as obsessive-compulsive symptoms rated by parents (57.0 *vs.* 45.33; p = 0.008, Table **2**). Females also scored greater than males in self-reported performance fears (66.4 *vs*. 51.0; p = 0.027) and separation anxiety (56.8 *vs*. 46.5; p = 0.038) (Table **2**).

Parent rated psychiatric symptoms and altered behaviors measured with the CBCL showed greater severity among females at internalizing problems (70.1 *vs.* 64.9; p = 0.016), somatic problems (66.3 *vs*. 61.23; p = 0.029), sluggish cognitive tempo (64.5 *vs*. 60.1; p = 0.026), obsessive-compulsive problems (67.7 *vs*. 62.2; p = 0.045) and post-traumatic stress problems (73.1 *vs*. 65.1; p = 0.006). Furthermore, the CBCL AAA index was significantly greater in females (207 *vs*. 189; p = 0.007), as significant was the difference between subscales summed up in its score, with anxious/depression score of 72.09 *vs*. 66.45 (p = 0.021), attention problems score of 67.4 *vs*. 61.7 (p = 0.015) and aggressive behaviors of 67.9 *vs*. 61.4 (p = 0.014) (Table **2**).

### 3.4 Multivariate Logistic Regression Model

To test for the weight in discriminating between males and females diagnosed with a major depressive episode with mixed features, items with a significantly different score between genders were put together in a multivariate logistic regression model. The model correctly predicted 78.5% of cases, classifying 86.0% of females and 63.6% of males.

Factors found to be significantly and independently more severe among females than males were: [a] excessive fatigue (OR 1.68; P = 0.025), [b] low self-esteem (OR 1.67; p = 0.04), [c] excessive weeping (OR 1.62; p = 0.021) measured by CDRS, and [d] severe mood dysregulation measured with the CBCL AAA index (OR 1.04; p = 0.015) (Table **3**).

As a result, in this model, experiencing higher excessive fatigue by one point increases the odds of identification of female mixed depression by 68% (OR = 1,68; 95% CI 1.07-2.65). Similar odds are found for low self-esteem (OR = 1.67; 95% CI 1.02-2.72) and excessive weeping (OR = 1.62; 95% CI 1.07-2.43). Higher scores in the CBCL AAA index increase the odds of identification of female mixed depression by 4% (OR = 1.04; 95% CI 1.01-1.07).

## 4 DISCUSSION

In our cohort of adolescents diagnosed with a major depressive episode with mixed features referred to a third-level child and adolescent psychiatry center, we found significant differences in overall symptom severity and specific psychopathological features between males and females. To our knowledge, this is the first study addressing sex differences between male and female adolescents diagnosed with a major depressive episode with mixed features.

The overall severity of depressive symptoms was quite high in our population, with an average score of depressive symptoms rated with the CDRS-R total score of 53.6, and more than 50% of subjects were diagnosed with “severe” depression and another 30% with “moderate” depression. Depression severity was significantly greater among women as rated by a statistically significant greater CDRS-R total score among women than men (56 *vs*. 48, p = 0.014, Table **1**) and by the classification of significantly more women in the moderate and severe depression group (33 *vs*. 24, p = 0.003, and 59 *vs*. 36, p = 0.003, respectively, Table **1**). Other studies on clinical samples of adolescents with depression, not selected for the presence of mixed features, failed to replicate the finding of higher severity of depressive symptoms in adult women with respect to men [19-23]. Regarding the interpretation of our findings, we suggest they represent a neurobiological, hormone, and psychosocial difference, as highlighted in recent literature; however, there may be a reporting bias [6].

The greater severity of depression found among female participants in our cohort appears to be coherent with the observed slightly higher hospitalization rate among females (19% *vs*. 4%, p = 0.08) and with the parent reports of symptoms at CBCL showing greater scores at internalizing, somatic and post-traumatic stress subscales among women versus men. Depressive symptoms difference did not appear in the CDI self and parent report, probably for underpowered samples.

Regarding specific dimensions of affective psychopathology, females scored significantly greater in several symptoms encompassing the core symptomatology of depression, including excessive weeping, excessive fatigue, low self-esteem, depressive feelings, and suicidal ideation. Some manic symptoms, including increased motor activity and pressured speech, were meanly greater among males.

To assess which symptoms best-distinguished females from males, controlling for the effects of the others, we tested single items of the scales in the logistic regression model, which yielded a very good fit to the data. The model correctly identified 86.0% of the girls and 63.6% of the boys, showing higher accuracy in classifying males compared to a previous study on depressed adolescents with similar methods [19]. The most distinctive features found to be significantly and independently associated with the female sex were higher levels of excessive fatigue and weeping, low self-esteem, and a higher CBCL AAA index (Table **3**). Clinicians rated depressive feelings and suicidal ideation, and parent-rated internalizing and anxious/depression symptoms items were higher in females, even if not significant in multivariate analysis. The first noticeable observation of our findings is that in this population, sex differences encompass the core affective dimension more than somato-autonomic dimensions, in contrast with studies on adult depression [6, 15, 17]. It can be concluded that girls with mixed depression have more explicit cognitive access to depressive feelings, which are reported in vivid details, and self-image is profoundly affected.

Differently from previous studies, in our cohort, “excessive weeping” is markedly different between sexes, representing a marker of lability in girls with mixed depression. We also highlight this item because this dimension is included in mixed features measured by the Koukopoulos Mixed Depression Rating Scale [47]. Of note, a study on adult inpatients [48] found more weeping together with lability in women compared to men showing more affective rigidity. Excessive fatigue is also distinctive for the female depressive profile, and this was reported to be a marker of severity and persistence of depressive symptomatology in community studies on depression [49].

Suicidal ideation was present in most of our adolescents and was found to be more severe in females (Table **2**); while suicidal behaviors were not uncommon in our cohort, sex difference was not significative, possibly for underpowered samples.

Overall, a more profound expression of core affective symptoms depicting a highly severe phenotype characterizes female mixed depression, with higher expression of dimensions warranting thoughtful clinical attention, such as fatigue, emotional lability, negative self-image, and suicidal thoughts.

Moreover, the CBCL AAA index, a measure of emotional dysregulation, was higher in females. Mean scores were higher than the first standard deviation described in the general population in both groups [45]. This represents a further marker of greater severity. It is worth noting that irritability, one of the most distinctive symptoms of depression in youth, was not reported to be different among genders, as revealed by clinician-administered scales (CDRS and KMRS), as well as ARI self and parent report, an instrument specifically designed to inquire irritability.

This finding differs from studies conducted on adults that found more irritability in women but indicated that if irritability was included as an additional diagnostic criterion for depression, the lifetime prevalence of depression increased more in men than women [50]. Other studies on adults did not find differences in irritability among adults with depression [51, 52], but some suggested that irritability may be distinctive in adult men with depression [53, 54]. Irritability, marked mood reactivity, and lack of psychomotor retardation, all of which are distinctive features of agitated depression among adults [47], are, in our experience, quite uniform among adolescent depression, except for bipolar depression, which has not been considered in this cohort. In our experience, contrary to what was reported by other groups [27], gender differences are present and are identified by CDRS and CBCL emotional dysregulation index more than KMRS, possibly matching the Koukopoulos criteria for Mixed Depression [47], involving the variable of a description of suffering or spells of weeping, but also encompassing excessive fatigue.

Studies on depression in adults report a considerable sex difference for atypical features of depression, which are more common among women [6]. Moreover, studies on gender differences in depression among adults recourse to the construct of “neuroticism” to explain higher sensitivity to negative affect, rumination and anxiety in females. The profile highlighted in our population seems not to endorse this model completely. Symptoms differences, instead, seem compatible with differences in specific domains of rumination and not with rumination tendency overall [55]. Our profile of female mixed depression reflects more dimensions of self-surveillance and negative body image [56], central for adolescent girls, more than “excessive guilt” and other negative attributional style correlates [57].

However, based on our findings, when a homogeneous population defined by the narrowest definition of mixed features according to DSM 5 is selected, sex differences encompass the focal dimensions of mixed depression of negative affect and its regulation with points of contact with Koukopoulos formulation of mixed depression, even if not fully recapitulating agitated depression of adults.

The difference in depressive symptoms between male and female adolescents was also found by Bennett and colleagues [19], who described more guilty, depressive and failure feelings, self-image dissatisfaction, more severe fatigue and lower school performance in females. The population included in their study was very different, the inclusion criteria were based on Spitzer RDC, and irritable depression was excluded, while Depression Not Otherwise Specified was included, representing a sample with overall lower severity of psychopathology, not comparable with our cohort, but sharing some findings with our results.

Females also showed greater scores for self- and parent-rated comorbid anxiety symptoms than males, including self-rated separation and performance fears, and greater scores for parent-rated obsessive-compulsive symptoms. More obsessions/compulsions in MASC 2 subscale in females is compatible with literature on OCD incidence differences between sexes in adolescence [58]. We did not find substantial inconsistencies between clinician and self-administered scales, which were found in contrast in other adolescents [19] and adult [59] studies.

Furthermore, different from studies on adults with unipolar [15] or bipolar [60] mood disorders, a significant difference in comorbidity profiles was not found. We might infer differences in comorbidities that come later with age, resulting from a more prolonged trajectory, a course upon which intervening is needed and early diagnosing depression in adolescence is valuable.

## 5 LIMITATIONS

A strength of our cohort is the homogeneity and accurate selection of patients with DSM-5 depression with mixed features by clinical consensus between evaluators. All participants were also characterized by the presence of three or more hypomanic symptoms measured by three or more scores of clinical relevance in KMRS items, reproducing a rigorous method used recently by other authors [27] to better define their cohorts with adolescent depression with mixed features as a unique constellation with a high level of complication. A limitation to consider is inherent to the study protocol, consisting of a retrospective collection of data on young patients, lacking a long-term follow-up. This may limit the diagnostic accuracy of BP I, BP II and BP NOS diagnoses since it is known that the stability of these diagnoses may vary with the length of longitudinal follow-up of children and adolescents and the consequent occurrence of further mood episodes. On the one hand, some patients presenting with a first depressive episode, particularly if presenting hypomanic features, may convert to bipolar disorder at follow-up [61], and patients with BP NOS and BP II may convert to BP I [62]. On the other hand, developmentally limited forms of bipolar disorder have been described [63]. Another possible limitation is the age difference between sexes at evaluation, albeit comparable age at first mood symptoms, indicating the longer duration of psychopathology in females at evaluation, possibly due to later referral or longer duration of subthreshold symptoms before referral to a third-level psychiatric service. A further limitation to consider is that our sample is selected from patients referred to a third-level center, which may not be representative of the general population.

## CONCLUSION

Female adolescents diagnosed with a depressive episode with DSM-5 mixed features are characterized by more overall severity of depressive symptoms, encompassing core affective dimensions (depressive feelings, low self-esteem) and excessive fatigue more than other somatic autonomic dimensions. Mixed features identified by KMRS are slightly higher in males, while females are possibly subtended by more emotional dysregulation at the CBCL AAA index and more excessive weeping. Although no difference has been found in DSM-5 syndromic comorbidities, more anxiety symptoms are reported by females. We suggest more sensible diagnostic instruments are needed to be developed to identify the widest spectrum of counterpolar mixed features in females.

## Figures and Tables

**Table 1:** Descriptive characteristics of the 76 study patients.

**Measure**	**All Subjects** **(n=76)**	**Males** **(n=25)**	**Females** **(n=51)**	***p*-value (Statistic)**
**Ages, Years (Mean ± SD)**				
At first major affective episode At evaluation	11.97 ± 3.36 14.48 ± 2.79	11.83 ± 3.85 13.45 ± 3.36	12.02 ± 3.18 14.9 ± 2.32	0.842 (-0.20) 0.022 (-2.35)
**Diagnosis (%, n)**				
Bipolar disorder Major depressive disorder Persistent major depressive disorder	32.9% (25) 57.9% (45) 9.2% (7)	40.0% (10) 48.0% (12) 12.0% (3)	29.4% (15) 62.7% (32) 7.80% (4)	0.422 (3.88)
**Level of Severity (%, n)**				
Mild CDRS-R < 45 Moderate CDRS-R 45-55 Severe CDRS-R > 55	18.4% (14) 30.3% (23) 51.3% (39)	40.0% (10) 24.0% (6) 36.0% (9)	7.8% (4) 33.3% (17) 58.8% (30)	0.003 (11.60)
**Co-Morbidity (%, n)**				
ADHD Anxiety disorders Oppositional defiant disorder Obsessive-compulsive disorder Tic disorders Eating disorders Learning disorder Substance abuse	5.3% (4) 25% (19) 2.6% (2) 2.6% (2) 2.6% (2) 6.57% (5) 10.52% (8) 6.57% (5)	8% (2) 32% (8) 4% (1) 4% (1) 0 12.0% (3) 16.0% (4) 4.0% (1)	3.9% (2) 21.6% (11) 2% (1) 2% (1) 3.9% (2) 3.92% (2) 7.84% (4) 7.84% (4)	0.59 (0.560) 0.40 (0.974) 0.60 (0.272) 0.60 (0.272) 0.31 (1.006) 0.324 (1.781) 0.427 (1.185) 0.465 (0.403)
**Self-Harm Status (%, n)**				
C-SSRS screening total score (mean±SD) Suicidal ideation ever Suicide attempters ever NSSI ever	2.09 ± 2.02 60.5% (46) 19.7% (15) 47.4% (36)	1.63 ± 2.13 52.0% (13) 8.0% (2) 36.0% (9)	2.25 ± 2.01 64.7% (33) 25.5% (13) 52.9% (27)	0.481 (-0.728) 0.287 (1.134) 0.072 (3.240) 0.164 (1.931)
**Clinician-Rated Scales (mean ± SD)**				
Depression (CDRS-R) Mania (K-MRS) Clinical Mania (K-MRS > 12)	53.61 ± 13.52 11.89 ± 3.86 46.05% (35)	48.24 ± 13.75 13.12 ± 4.74 60% (15)	56.24 ± 12.73 11.29 ± 3.23 39.22% (20)	0.014 (-2.505) 0.052 (1.977) 0.088 (2.917)
**Self-Rated Scales (mean ± SD)**				
Anxiety (MASC-2) Depression (CDI-2) Irritability (ARI)	62.77 ± 14.03 75.27 ± 15.91 7.88 ± 3.86	55.17 ± 13.99 73.17 ± 18.61 8.11 ± 4.41	65.63 ± 13.36 76.06 ± 15.41 7.78 ± 3.72	0.122 (-1.616) 0.714 (-0.372) 0.833 (0.213)
**Parent Report Scales (mean ± SD)**				
Behavioral (CBCL total) Mood dysregulation (CBCL AAA) Anxiety (MASC-2) Depression (CDI-2) Irritability (ARI)	30.87 ± 6.22 201.29 ± 25.66 65.76 ± 13.19 67.25 ± 8.68 6.75 ± 4.06	31.50 ± 3.11 189.55 ± 22.56 56.83 ± 9.54 65.83 ± 11.39 5.33 ± 2.92	30.64 ± 7.15 207.30 ± 25.29 69.33 ± 12.97 67.86 ± 7.67 7.30 ± 4.36	0.822 (0.230) 0.007 (-2.774) 0.047 (-2.129) 0.645 (-0.468) 0.223(-1.244)
**Psychiatric Hospitalization**				
Ever hospitalized (%, n) Admissions/person (mean±SD)	14.47% (11) 0.34 ± 1.05	4.0% (1) 0.08 ± 0.40	19.61% (10) 0.47 ± 1.24	0.089 (3.301) 0.129 (-1.533)

**Statistics**: Results are presented as percentages and numbers (%, n) for categorical variables and as mean and standard deviation (mean±SD) for continuous variables. Univariate analyses have been carried out with unpaired t-test for continuous and chi-square for categorical measures. Statistic value (t or chi-square as appropriate) has been reported in brackets in the p-value column.

**Abbreviations**: ADHD= Attention Deficit Hyperactivity Disorder; CBCL= Children Behavior Check-List; AAA= Aggression/Anxiety-Depression/Attention index.

**Note**: calculated by summing the scores for attention problems, aggression and anxious-depressed syndromal scales; “Ever hospitalized” refers to the rate of patients who have had at least one inpatient admission in the psychiatry unit lifetime; “Admission/person” refers to the mean number of inpatient admissions in the psychiatry unit lifetime.

**Table 2:** Psychopathological features in males *versus* females (n=76 patients).

**Measure**	**All Subjects ** **(n=76)**	**Males** **(n=25)**	**Females** **(n=51)**	***p*-value (Unpaired *t*)**
**Mania Symptoms (K-MRS)**				
Pressured speech	1.42 ± 0.82	1.80 ± 1.16	1.24 ± 0.513	0.004 (2.960)
Increased motor activity	1.83 ± 0.9	2.12 ± 0.88	1.69 ± 0.88	0.048 (2.013)
K-MRS total score	11.89 ± 3.86	13.12 ± 4.74	11.29 ± 3.23	0.052 (1.977)
**Depressive Symptoms (CDRS-R)**				
Excessive weeping	3.26 ± 1.96	2.20 ± 1.44	3.78 ± 1.98	0.001 (-3.555)
Excessive fatigue	3.55 ± 1.68	2.76 ± 1.69	3.94 ± 1.54	0.003 (-3.040)
Low self-esteem	4.21 ± 1.59	3.60 ± 1.38	4.51 ± 1.61	0.014 (-2.55)
CDRS-R total score	53.61 ± 13.52	48.24 ± 13.75	56.24 ± 12.73	0.014 (-2.505)
Depressive feeling	4.43 ± 1.60	3.80 ± 1.58	4.75 ± 1.53	0.015 (-2.498)
Suicidal ideation	2.84 ± 1.80	2.2 ± 1.35	3.16 ± 1.92	0.029 (-2.498)
**Self-Rated Symptoms (CBCL)**				
Post-traumatic stress problems	70.30 ± 11.64	65.05 ± 10.21	73.05 ± 11.50	0.006 (-2.848)
CBCL AAA index	201.29 ± 25.66	189.55 ± 22.56	207.30 ± 25.29	0.006 (-2.880)
Attention problems	65.43 ± 9.73	61.68 ± 7.72	67.35 ± 10.16	0.015 (-2.506)
Internalizing problems	68.06 ± 10.43	64.09 ± 8.19	70.09 ± 10.96	0.016 (-2.482)
Anxious/depression	70.18 ± 10.55	66.45 ± 7.66	72.09 ± 11.37	0.021 (-2.366)
Sluggish cognitive tempo	62.89 ± 7.75	60.05 ± 6.85	64.45 ± 7.84	0.026 (-2.299)
Somatic Problems	64.58 ± 9.99	61.23 ± 7.25	66.33 ± 10.83	0.029 (-2.243)
Obsessive-compulsive problems	65.83 ± 11.07	62.18 ± 9.59	67.74 ± 11.42	0.045 (-2.059)
**Self-Rated Anxiety (MASC-2)**				
Obsessive-compulsive (parent report)	53.67 ± 12.33	45.33 ± 4.97	57.0 ± 12.91	0.008 (-2.991)
Performance fears (self-report)	61.75 ± 12.81	51.0 ± 12.29	66.36 ± 10.26	0.027 (-2.685)
Separation anxiety (self-report)	53.95 ± 10.49	46.50 ± 4.42	56.75 ± 10.83	0.038 (-2.222)
Parent report tot.	65.76 ± 13.19	56.83 ± 9.54	69.33 ± 12.97	0.047 (-2.129)

**Note**: For each test, the total score has been reported. Single items scores for CDRS-R and K-MRS were reported, if significantly different between the sexes. CBCL and MASC-2 scale scores were also reported to be significantly different between the sexes. Continuous variables are presented as mean ± standard deviation. Univariate analyses were performed with unpaired *t*-tests for continuous variables, and the t-value is reported in brackets in the p-value column.

**Abbreviations**: AAA= Aggression/Anxiety-Depression/Attention index, calculated by summing the scores for attention problems, aggression and anxious-depressed syndromal scales. ADHD= Attention-Deficit / Hyperactivity Disorder; C-SSRS= Columbia Suicide Severity Rating Scale; CBCL= Child Behavior Checklist; KMRS=Kiddie-SADS Mania Rating Scale; K-SADS-PL= Kiddie-Schedule for Affective Disorders and Schizophrenia for School-aged Children, Present and Lifetime.

**Table 3:** Logistic regression model (n=76 patients).

**Measure**	**B Coefficients ** **(Standard Error)**	**Odds Ratio** **(95% Confidence Interval)**	**Chi-Square**	**Significance ** **(Wald Test)**
CBCL AAA index	0.04 (0.02)	1.04 (1.01-1.07)	7.54	0.015 (5.89)
CDRS-R Excessive Weeping	0.48 (0.21)	1.62 (1.07-2.43)	6.21	0.021 (5.31)
CDRS-R Excessive Fatigue	0.52 (0.23)	1.68 (1.07-2.65)	5.95	0.025 (5.06)
CDRS-R Low Self-esteem	0.51(0.25)	1.67 (1.02-2.72)	4.83	0.040 (4.21)
KMRS Increased Motor Activity	-	-	0.01	0.949
KMRS Pressured Speech	-	-	2.38	0.123
CDRS-R Total Score	-	-	3.39	0.066
CDRS-R Depressive Feeling	-	-	3.09	0.079
CDRS Suicidal Ideation	-	-	0.35	0.552

**Statistics**: Multinomial logistic regression model. Predicted variable: female sex. Dependent variables not significantly associated with the female sex (*p* ≥ 0.05) were KMRS pressured speech, KMRS increased motor activity, CDRS-R total score, CDRS-R depressive feeling, and CDRS suicidal ideation.

## Data Availability

Not applicable.

## LIST OF ABBREVIATIONS

ADHD: Attention-Deficit/Hyperactivity Disorder
ARI: Affective Reactivity Index
C-SSRS: Columbia Suicide Severity Rating Scale
CBCL: Child Behavior Checklist
CDI: Child Depression Inventory
CDRS-R: Children Depression Rating Scale-Revised
CGAS: Clinical Global Assessment Scale
DP: Dysregulation Profile
K-SADS-PL: Kiddie-Schedule for Affective Disorders and Schizophrenia for School-aged Children, Present and Lifetime
KMRS: Kiddie-SADS Mania Rating Scale
MASC 2: Multidimensional Anxiety Scale for Children 2
MDE: Major Depressive Episode
NSSI: Non-Suicidal Self-Injury
